# Characterisation of inflammatory response, coagulation, and radiological findings in Katayama fever: a report of three cases at the Medical University of Vienna, Austria

**DOI:** 10.1186/1471-2334-14-357

**Published:** 2014-07-01

**Authors:** Heimo Lagler, Cihan Ay, Fredrik Waneck, Rainer Gattringer, Wolfgang Graninger, Michael Ramharter

**Affiliations:** 1 Department of Medicine I, Division of Infectious Diseases and Tropical Medicine, Medical University of Vienna, Währinger Gürtel 18-20, Vienna 1090, Austria; 2 Department of Medicine I, Division of Haematology and Haemostasiology, Medical, University of Vienna, Währinger Gürtel 18-20, Vienna 1090, Austria; 3 Departement of Biomedical Imaging and Image-guided Therapy, Division of Cardiovascular and Interventional Radiology, Medical University of Vienna, Währinger Gürtel 18-20, Vienna 1090, Austria; 4 Institute of Hygiene, Microbiology and Tropical Medicine, Elisabethinen Hospital Linz, Fadingerstrasse 1, Linz 4020, Austria; 5 Institut für Tropenmedizin, University of Tübingen, Wilhelmstraße 27, Tübingen 72074, Germany

**Keywords:** Schistosomiasis, Katayama, Inflammation, Coagulation, Radiology, Africa

## Abstract

**Background:**

Katayama fever is an acute clinical condition characterised by high fever, dry cough and general malaise occurring during early *Schistosoma* spp*.* infection. It is predominantly reported in travellers from non-endemic regions. Whereas the immunological response to *Schistosoma* infection is well characterised, alterations in inflammatory markers and coagulation in response to acute infection are poorly understood.

**Methods:**

Here we report the clinical, laboratory and radiological characteristics of three returning travellers with Katayama fever. Inflammatory markers and coagulation status were assessed repeatedly during follow-up to characterise the host response to infection. Radiographic findings were correlated with clinical and laboratory markers.

**Results:**

Clinical symptoms were suggestive of a significant inflammatory response in all patients including high fever (>39°C), cough, and general malaise. Classical inflammatory markers including blood sedimentation rate, C-reactive protein, and serum amyloid A were only moderately elevated. Marked eosinophilia (33–42% of white blood cells) was observed and persisted despite anti-inflammatory and anthelminthic treatment for up to 32 weeks. Analysis of blood coagulation markers indicated increased coagulability reflected by elevated D-dimer values (0.57–1.17 μg/ml) and high thrombin generating potentials (peak thrombin activity: 311–384 nM). One patient showed particularly high levels of microparticle-associated tissue factor activity at initial presentation (1.64 pg/ml). Multiple pulmonary and hepatic opacities demonstrated by computed tomography (CT) scanning were associated with raised inflammatory markers in one patient.

**Conclusions:**

The characterisation of the inflammatory response, blood coagulation parameters and radiological findings in three patients adds to our current understanding of Katayama fever and serves as a starting point for further systematic investigations of the pathophysiology of this acute helminthic infection.

## Background

Schistosomiasis is a debilitating chronic infection caused by trematode worms of the genus *Schistosoma*. Rural regions in the tropics are most affected leading to a global disease burden of 3–70 million disability-adjusted life years lost [1]. Humans become infected during freshwater exposure by larval stages of *Schistosoma* spp. (cercariae) shed from the snail intermediate host. Cercariae penetrate the intact skin, migrate to and evolve in the venous drainage systems of several tissues in the human host, including the urogenital tract (*S. haematobium*) and the intestines (*S. mansoni, intercalatum, japonicum, mekongi*). The presence of adult worms and their eggs in human tissues ultimately lead to a variety of chronic pathologies including haematuria, haematochaezia, liver fibrosis, bladder cancer, and chronic inflammation of the urogenital tract [2].

Katayama fever is an acute inflammatory syndrome resulting in high fever, cough and general malaise, occurring 3–8 weeks after infection by cercariae, that has chiefly been described in travellers from non-endemic regions [3]. The clinical symptoms of Katayama fever are mediated by a hypersensitivity reaction to the intravascular trematodes. Acute clinical symptoms gradually subside even without specific anthelmintic treatment and are superseded by chronic granulomatous inflammation which causes the vast majority of morbidity.

Although several studies have investigated the immunological aspects of chronic *Schistosoma* infection in animal models and humans, little is known about inflammatory markers and radiological signs of inflammation in Katayama fever [4]. No data describing the consequences of intravascular helminth migration on the activation of blood coagulation mechanisms in Katayama fever have been published so far. Here we report the clinical course, radiological findings, and observed alterations in markers of inflammation and blood coagulation in three returning travellers suffering from Katayama fever.

## Methods

Patients were initially assessed at three different hospitals in Eastern Austria. Two patients were subsequently hospitalized at the Division of Infectious Diseases and Tropical Medicine at the Medical University of Vienna, and the third patient was hospitalized at a referral hospital in the province of Upper Austria. Written informed consent was obtained from patients for additional blood sampling for the analysis of coagulation parameters and for permission to analyse clinical data. Ethical clearance was waived for this case series by the institutional review board of the Medica University of Vienna. Radiological evaluation of patients was performed at the referring hospitals. D-dimer, *in-vitro* thrombin generation potential and microparticle-associated tissue factor (MP-TF) activity measurements were performed at the Medical University of Vienna using previously validated assays [5,6]. Inflammatory markers were assessed using internally and externally quality-controlled standard methods. Data were captured in an electronic database; due to the small sample size, original data were reported and non-parametric summary measures were presented as appropriate.

## Results

### Clinical presentations of patients and radiological findings

Two female and one male Austrian citizen aged 19–21 years all presented at hospitals in Eastern Austria with an abrupt onset of general malaise, high fever and a productive cough. All patients had returned from a four-week trip to the Republic of Tanzania three weeks prior to the onset of symptoms. The journey consisted of work in a developmental aid programme in a rural community, a game park visit and freshwater exposure in Lake Malawi. On admission, they were all treated empirically with broad-spectrum beta-lactam antibiotics for suspected pneumonia. The two female patients were subsequently transferred to the inpatient ward of the Department of Infectious Diseases at the Medical University of Vienna due to progressive clinical deterioration. The male patient was hospitalized at a referral hospital in the province of Upper Austria.

**Figure 1:**
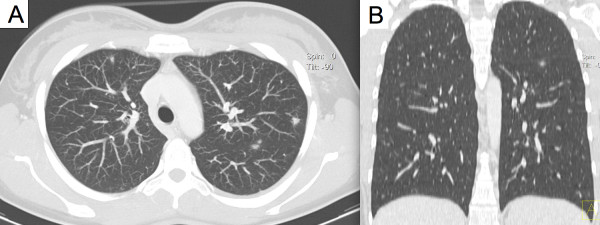
Radiographic findings of lung computed tomography during Katayama syndrome (axial (A) and coronal (B) reconstruction).

**Figure 2:**
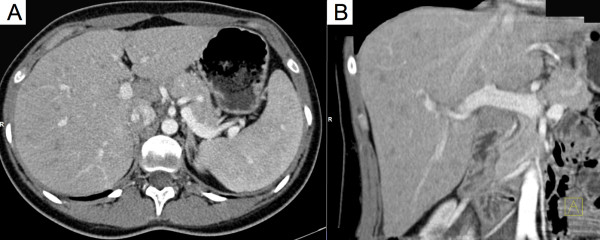
Radiographic findings of abdominal computed tomography during Katayama syndrome (axial (A) and coronal (B) reconstruction).

On referral, the diagnosis of Katayama fever was made based on the clinical history, laboratory parameters and radiological findings. Anti-inflammatory treatment with corticosteroids and concomitant anthelminthic treatment with praziquantel were administered. Patients responded rapidly to this treatment and were discharged within five days. Malaria and systemic bacterial or fungal infections were excluded with serial, negative thick blood smears and blood cultures. Out-patient follow-up visits were performed over a six month period. *Schistosoma-*specific antibody titres – initially negative during the acute phase of infection – later converted to a positive result in all three patients and confirmed the initial clinical diagnosis. No urinary/faecal excretion of *Schistosoma* eggs was detected during follow-up in any of the patients. A second cycle of praziquantel treatment was administered three months after initial presentation to target previously immature stages, which are less susceptible to praziquantel.Chest X-rays in all patients did not show pathological changes. A slightly enlarged spleen on abdominal ultrasound was documented in one case (14.4 cm diameter). In one patient, thoracic and abdominal CT was performed at the referring hospital due to the clinical diagnosis of systemic inflammatory response syndrome. This revealed multiple, bilateral pulmonary opacities measuring up to 9 mm (Figure 1), and hypodense foci in the liver (Figure 2). These radiological findings were initially reported as being consistent with numerous septic abscesses or metastatic malignancy.

### Characterisation of inflammatory response and blood coagulation parameters

Clinical and laboratory markers of inflammation were repeatedly assessed during follow-up of patients (Table 1) All individuals experienced high fever >39°C at initial presentation. Classical markers of inflammation including C-reactive protein (CRP; range: 6–23 mg/L), serum amyloid A (SAA; 52–67 mg/L), and blood sedimentation rate (BSR; 27/36–82/110 mm/h) were moderately elevated. Correspondingly, the total leukocyte count was elevated, with a marked eosinophilia ranging between 33-42% of white blood cells observed in all patients. Inflammatory markers rapidly returned to normal levels after the initiation of anti-inflammatory treatment with corticosteroids; however, eosinophilia only gradually declined and persisted for up to 32 weeks after the initiation of treatment. Elevated lactate dehydrogenase (LDH) levels were present in one patient with a particularly pronounced inflammatory response (391 U/L).

**Table 1:** Clinical and laboratory markers of inflammation at initial presentation and during follow-up

**Parameter**	**Patient**	**Baseline**	**Week 1**	**Week 2**	**Week 6**	**Week 11**	**Week 32**
Temperature (°C)	A	max 39.0	<37	<37	<37	<37	<37
	B	max 39.6	<37	<37	<37	<37	<37
	C	max 39.3	<37	<37	<37	<37	<37
BSR (mm)	A	55/82	-	31/60	7/22	12/22	12/20
	B	82/110	-	64/102	12/28	14/31	15/31
	C	27/36	25/30	-	-	-	-
CRP (<10 mg/L)	A	6	3	0.7	0.3	0.4	0.2
	B	23	10	6	1	1	2
	C	23	11	-	-	-	-
PCT (<0.5 ng/ml)	A	0.13	-	-	-	-	-
	B	0.17	-	-	-	-	-
	C	-	-	-	-	-	-
SAA (<5 mg/L)	A	52	32	-	-	-	-
	B	67	31	-	-	-	-
	C	-	-	-	-	-	-
LDH (<247 U/L)	A	192	173	206	189	149	157
	B	391	237	275	204	180	183
	C	200	202	-	-	-	-
Leukocyte count (4–10 G/l)	A	5.9	8.5	2	5.4	4.7	5.2
	B	16.1	17.1	14.2	6.7	6.5	7.6
	C	12.7	11.9	-	-	-	-
Eosinophil count (0–0.4 G/l)	A	2	3.1	1.2	0.4	0.3	0.2
	B	6.7	7.2	5.8	0.8	0.7	0.2
	C	4.5	4	-	-	-	-
Eosinophil relative (0-4%)	A	33	36	15.1	7.4	6.0	3.8
	B	42	42	41	12	16	2.6
	C	35	33.5	-	-	-	-

max: maximum; BSR: blood sedimentation rat after one hour/after two hours; CRP: C-reactive protein; PCT: procalcitonin; SAA: serum amyloid A; LDH: lactate dehydrogenase.

Blood coagulation parameters were assessed in the two female patients (Table 2). Here, D-dimer levels were elevated at presentation indicating activation of haemostasis and fibrinolysis (1.2 and 0.6 μg/mL, respectively), and fibrinogen levels were slightly increased (477–517 mg/L). The thrombin generating potential was increased at presentation as reflected by a higher peak measurements (384 and 311 nM, respectively) compared to follow-up. In one patient the peak thrombin generating potential was increased at the end of follow-up without evidence for disease activity. Finally, the MP-TF activity was markedly raised in one patient at 1.64 pg/mL, before gradually declining to 0.03 pg/mL.

**Table 2:** Parameters of coagulation cascade at initial presentation and during follow up

**Parameter**	**Patient**	**Baseline**	**Week 1**	**Week 6**	**Week 11**	**Week 32**
D-dimer (<0.5 μg/ml)*	A	0.57	0.06			0.28
	B	1.17	1.21			0.46
Fibrinogen (180–390 mg/dl)	A	477	443	331	302	312
	B	517	369	307	281	341
Peak thrombin generation (nM)	A	311	-	190	169	122
	B	384	-	202	237	506
MP-TF activity (pg/ml)	A	0.09	-	0.05	0	0.85
	B	1.64	-	1.10	0.12	0.03

*this cut-off indicates non-increased or negative D-dimer values.

MP-TF = microparticle-associated tissue factor.

## Discussion

Katayama fever is thought to be mediated by a systemic hypersensitivity reaction to migrating parasites and circulating immune complexes at the onset of egg production [4], leading to the classical clinical symptoms. The clinical presentation of the reported cases was indicative of such an inflammatory response - patients had a fever >39°C and were significantly incapacitated by the clinical disease course. Contrary to these findings, however, inflammatory markers including CRP and BSR were only modestly elevated. At the same time eosinophilia – a hallmark of invasive helminthic infections – was markedly elevated. Interestingly, eosinophilia persisted for more than six months prior to normalisation indicating prolonged exposure to helminthic antigen stimulation. This finding may be explained by the fact that praziquantel – although already administered in our patients during acute infection – has little activity against the early developmental stages of schistosomal worms and complete cure from all intravascular worms was therefore achieved only after re-administration of praziquantel during the follow up period [7].

Distinct radiographic abnormalities of the lungs in Katayama fever, including patchy pulmonary infiltrates on chest X-ray and single/multiple pulmonary nodules with ground-glass halos on CT, have been previously described [8-10]. However, radiological alterations in other viscera have been less well described. CT in one of our patients showed radiological alterations of the lung parenchyma consistent with previous reports. In addition, multiple hypodense foci were demonstrated in the liver supporting the hypothesis that Katayama fever represents are more generalized inflammatory response. Whereas these findings are useful from a scientific point of view, the authors are convinced that CT should not be considered as a standard diagnostic examination for patients with suspected Katayama fever, given that the CT features are not pathognomonic of this disease and the risks of exposure to radiation most likely outweigh the diagnostic benefit.

Detailed analysis of coagulation mechanisms including: D-dimer, which indicates an activation of haemostasis and fibrinolysis; the thrombin generation potential, a global in-vitro assay indicating an individual’s coagulation potential; and measurement of the MP-TF activity, which reflect a prothrombotic state, showed considerable variability in these cases. One may speculate that the intravascular migration of helminths would result in activation of the coagulation cascade; this was demonstrated by increased levels of D-dimer, a high peak thrombin and a marked increase of the MP-TF activity in one patient. However, these features were less evident in a second patient, explained either by a less pronounced response in this patient or that the peak in alterations may have been missed during the referral from another hospital. Despite the intravascular localisation of schistosomal worms and the activation of coagulation mechanisms, clinical complications such as thrombotic events are not commonly reported in the context of acute schistosomiasis, consistent with our cases. A better understanding of the underlying mechanisms for this discordance of clinical and laboratory findings may improve our knowledge of the complex interplay between helminth pathogens and the host’s response.

## Conclusion

The characterisation of the inflammatory response, coagulation parameters and description of corresponding radiographic findings in our patients may provide helpful information for the diagnostic workup of future patients with acute febrile conditions returning from the tropics. Based on these preliminary findings, a further systematic evaluation of the impact of intravascular helminth infection on blood coagulation and the inflammatory response in a larger, case-controlled study may be warranted.

## Abbreviations

MP-TF: Microparticle-associated tissue factor; CRP: C-reactive protein; SAA: serum amyloid A; BSR: blood sedimentation rate; LDH: lactate dehydrogenase; CT: computed tomography; max: maximum; PCT: Procalcitonin.

## Competing interests

The authors declare that they have no competing interests.

## Authors’ contributions

HL, RG, MR collected, assembled and analysed the data. CA analysed, supervised and interpreted all blood coagulation parameters. FA supervised and interpreted all CT scan findings. HL, WG and MR contributed to the study design, interpretation of data, writing and revision of the article. All authors read and approved the final manuscript.

## Pre-publication history

The pre-publication history for this paper can be accessed here:

http://www.biomedcentral.com/1471-2334/14/357/prepub

## References

[B1] King CH Dangerfield-Cha M The unacknowledged impact of chronic schistosomiasis Chronic Illn 2008 14 1 65 79 18322031 10.1177/1742395307084407

[B2] Gryseels B Polman K Clerinx J Kestens L Human schistosomiasis Lancet 2006 14 9541 1106 1118 16997665 10.1016/S0140-6736(06)69440-3

[B3] Doherty JF Moody AH Wright SG Katayama fever: an acute manifestation of schistosomiasis BMJ 1996 14 7064 1071 1072 8898604 10.1136/bmj.313.7064.1071 PMC2352353

[B4] Ross AG Vickers D Olds GR Shah SM McManus DP Katayama syndrome Lancet Infect Dis 2007 14 3 218 224 17317603 10.1016/S1473-3099(07)70053-1

[B5] Ay L Kopp HP Brix JM Ay C Quehenberger P Schernthaner GH Pabinger I Schernthaner G Thrombin generation in morbid obesity: significant reduction after weight loss J Thromb Haemost 2010 14 4 759 765 20102484 10.1111/j.1538-7836.2010.03766.x

[B6] Thaler J Ay C Mackman N Bertina RM Kaider A Marosi C Key NS Barcel DA Scheithauer W Kornek G Zielinski C Pabinger I Microparticle-associated tissue factor activity, venous thromboembolism and mortality in pancreatic, gastric, colorectal and brain cancer patients J Thromb Haemost 2012 14 7 1363 1370 22520016 10.1111/j.1538-7836.2012.04754.x

[B7] Bottieau E Clerinx J de Vega MR Van den Enden E Colebunders R Van Esbroeck M Vervoort T Van Gompel A Van den Ende J Imported Katayama fever: clinical and biological features at presentation and during treatment J Infect 2006 14 5 339 345 16169593 10.1016/j.jinf.2005.07.022

[B8] Weber-Donat G Donat N Margery J Acute pulmonary schistosomiasis: computed tomography (CT) findings Am J Trop Med Hyg 2010 14 3 364 20207855 10.4269/ajtmh.2010.09-0425 PMC2829891

[B9] Soares Souza A Jr Marchiori E Maluf Cury P Gasparetto EL Escuissato DL Acute pulmonary schistosomiasis: correlation between the high-resolution CT and pathological findings Rev Port Pneumol 2007 14 5 741 744 17962893 10.1016/s2173-5115(07)70369-x

[B10] Voieta I Andrade LM Lambertucci JR Chest helical computed tomography scan shows pulmonary micronodules and condensation in acute schistosomiasis mansoni Rev Soc Bras Med Trop 2012 14 5 659 23152357 10.1590/s0037-86822012000500025

